# A Contemporary Review of Epidemiology, Risk Factors, Etiology, and Outcomes of Premature Stroke

**DOI:** 10.1007/s11883-022-01067-x

**Published:** 2022-11-14

**Authors:** Thomas B. H. Potter, Jonika Tannous, Farhaan S. Vahidy

**Affiliations:** 1 Department of Neurosurgery, Houston Methodist, Josie Roberts Administration Building, 4.123, 7550 Greenbriar Drive, Houston, TX 77030 USA; 2 Houston Methodist Neurological Institute, Houston Methodist, Houston, TX USA; 3 Department of Population Health Sciences, Weill Cornell Medicine, New York, NY USA; 4 Center for Health Data Science and Analytics, Houston Methodist, Houston, TX USA

**Keywords:** Ischemic stroke, Young stroke, Stroke epidemiology, Stroke etiology, Stroke outcomes, Intracerebral hemorrhage

## Abstract

**Purpose of Review:**

Recent data identifies increases in young ischemic and hemorrhagic strokes. We provide a contemporary overview of current literature on stroke among young patients or premature stroke along with directions for future investigation.

**Recent Findings:**

Strokes in the young are highly heterogenous and often cryptogenic. Sex distribution and risk factors shift from women among the youngest age groups (< 35) to men over the age of 45, with a coinciding rise in traditional vascular risk factors. Incidence is higher in minority and socioeconomically disadvantaged populations, and the impact of stroke among these communities may be exaggerated by disparities in symptom recognition and access to care. Special diagnostic work-up may be needed, and a lower threshold for diagnosis is warranted as potential misdiagnosis is a concern and may preclude necessary triage and management.

**Summary:**

Although “premature strokes” form a relatively small proportion of total incidence, they vary greatly across subgroups and present an outsized impact on quality of life and productivity.

## Introduction

Stroke (including ischemic and hemorrhagic phenotypes) remains the second leading cause of death globally and the third leading causing of death and disability [1]. Over the last two decades (1990–2019), there has been an increase in absolute number of incident strokes by 70%, prevalent strokes by 85%, deaths from stroke by 43%, and disability adjusted life-years (DALYs) by 32% [1]. Although a decline in age-standardized incidence rate has been reported, among people < 70 years both prevalence and incidence rates have increased by 22% and 15%, respectively. In addition to aggregate global data, emerging evidence of the rising burden of strokes among the young is also documented from national and regional analyses [2, 3•, 4••]. These so called “premature strokes” have a disproportionate societal burden, as they curtail productive years of life and predispose younger adults to longer-term sequalae and complications, including recurrent cerebrovascular events. We provide a contemporary review of the epidemiology, risk factors, etiological mechanisms, and outcomes of premature stroke. Though we broadly address all stroke sub-types, the review primarily focuses on ischemic strokes (IS), which constitute 87% and 63% of all stroke sub-types in the USA and globally, respectively.

## What Is Premature Stroke?

In the current literature, the terms “premature stroke,” “early-onset stroke,” and “stroke among the young/young stroke” have been used interchangeably. This is perhaps because there is no firm definition for what constitutes a premature stroke. Additionally, there is not a consistent definition of “young” or “juvenile” ages. While the lower age cutoff is fairly uniform at 18 years, upper limits span the decade between 45 and 55 years of age, with 45 and 50 representing the most frequently selected cutoffs. These cutoffs fall below the critical age of 55, after which stroke incidence doubles for each advancing decade of age [5]. Furthermore, they represent the lowest quartile of the overall stroke age distribution, the median of which is 60 to 65 years. To provide a more inclusive assessment, we will approach the topic through the broader age range of 18–55, which covers most relevant research.

## Epidemiology of Premature Stroke

As with older populations, IS typically accounts for the greatest proportion of incident strokes (44–65%) among younger patients, followed by intracerebral hemorrhage (ICH; 17–39%) and subarachnoid hemorrhage (SAH; 16–20%) [6, 7]. Rates vary considerably between cohorts, and a meta-analysis reports wide ranges for each subtype [8]. Overall, the incidence of stroke under 44 has increased from 5–17 per 100,000 person-years in the 1990s to 11–28 in more recent estimates [4••, 9, 10].

### Age and Sex

Young stroke accounts for 10–15% of total stroke patients. However, heterogeneity within the young is high and may present analytical challenges [11, 12]. Although convenient, treating the 18–55 age group as a monolith may overlook shifts in risk factors and etiologies at different stages of adulthood. Stroke incidence is age-specific even within the young, as occurrence rises exponentially across the 15–50 age range [13]. Therefore, it may be valuable to study epidemiology of stroke in the young in subgroups of 18–34, 35–45, and 46–55 years of age. While stroke within the 18–34 age range is uncommon [13], women have a 26–56% higher likelihood of pre-mature IS than men, depending on stroke subtype [14, 15]. From 35 years of age onwards, overall stroke incidence increases, with males (vs. females) at an increasingly higher stroke risk. This age-associated increase in stroke risk among males is largely attributable to traditional risk factors [12, 16, 17•]. Stroke subtype also seems to change with age. The proportion of lacunar and large artery strokes increases for patients over 40, while cardioembolic, cryptogenic, and “other” stroke types decrease [13, 18, 19]. In ICH populations, recent significant increases in incidence rates have been found for patients in the 18–44 and 45–64 age ranges over the past two decades [3•]. The effect modification of age and stroke risk by sex among the young has largely been reported from high-income countries. It is likely that varying patterns of differential stroke risk between males and females will be observed in global data [4••].

### Race and Ethnicity

Black and African American cohorts have 2–5 times higher stroke risk across various sub-types [20–23], with disparities appearing to be highest within the 35–44 age group. Specific increases have been noted for lacunar stroke, mediated by an increased prevalence of hypertension among African Americans [18]. These findings are noticeably linked to geography. Studies from Europe report overall similar trends, albeit to a lesser extent [24]. Studies from the Caribbean offer contrasting evidence, indicating that the increased prevalence found in Black and African American cohorts may stem primarily from socioeconomic and environmental variables [20]. Black patients who experience premature strokes show higher rates of hypertension, type II Diabetes, and congestive heart failure [25]. Similarly available evidence suggests that Hispanic cohorts have higher rates of stroke than non-Hispanic Whites [21, 26]. Overall, higher odds for IS are reported among both young racial minorities and Hispanic populations within the USA [27], although lower odds for transient ischemic attacks (TIAs) and IS have been documented specifically within the 20–24 age range [27].

### Geography

Extending outside of the USA, Boot et al. have provided a valuable perspective on global distribution of premature stroke. Generally, rates of young stroke appear to be consistent across North America, Australia, and Asia, at a rate of 20 per 100,000 person-years. This rate is slightly lower in European studies but approximately doubles among African cohorts [4••]. Although stroke prevalence is relatively higher, the overall rates of smoking within African countries have decreased over the past few decades [28, 29], and perspectives on how this may change the incidence of premature stroke are lacking. Compared to trends commonly found within overall US cohorts, trends among the young in Asian countries show increased proportions of large-vessel thrombosis and intra-cranial atherosclerosis [30–32]. Certain uncommon cerebrovascular conditions such as Moyamoya disease have been increasingly reported in Asian populations [33], although it is doubtful that Moyamoya alone accounts for the increasing incidence of stroke among the young. Finally, a recent study within a small Saudi Arabian cohort identified a high rate of dyslipidemia (71.4%) and small vessel occlusion (31.7%). While etiologies appear to have some differences across international cohorts, vascular risk factors do appear to be largely consistent, with multiple risk factors appearing in stroke patients [34]. This similar risk-factor profile provides a shared avenue for intervention and improvement of modifiable vascular risks as a priority for young populations worldwide.

## Etiology

Unlike older patients, etiologically most premature IS are classified as cryptogenic (24–53%), followed by those that may be due to cardio-embolism (10–34%), large artery atherosclerosis (4–29%), or small vessel disease (12–26%) [10, 13, 35, 36]. Cervicospinal dissection appears to be particularly prominent as well, causing up to 35% of IS among the young [37]. Among young cryptogenic strokes, patent foramen ovale (PFO) has been found in 40–56% of cases [38, 39] and patients showed high rates of hyperlipidemia, hormonal contraception, and migraine with aura, which may interact with PFO to increase cryptogenic stroke risk [40, 41]. Young ICH is most frequently linked with vascular malformation or hypertensive etiologies, depending on the cohort [42, 43]. Taken together, these findings show noteworthy differences between the causes and presentations of stroke between premature and older cohorts.

## Risk Factors

**Fig. 1:**
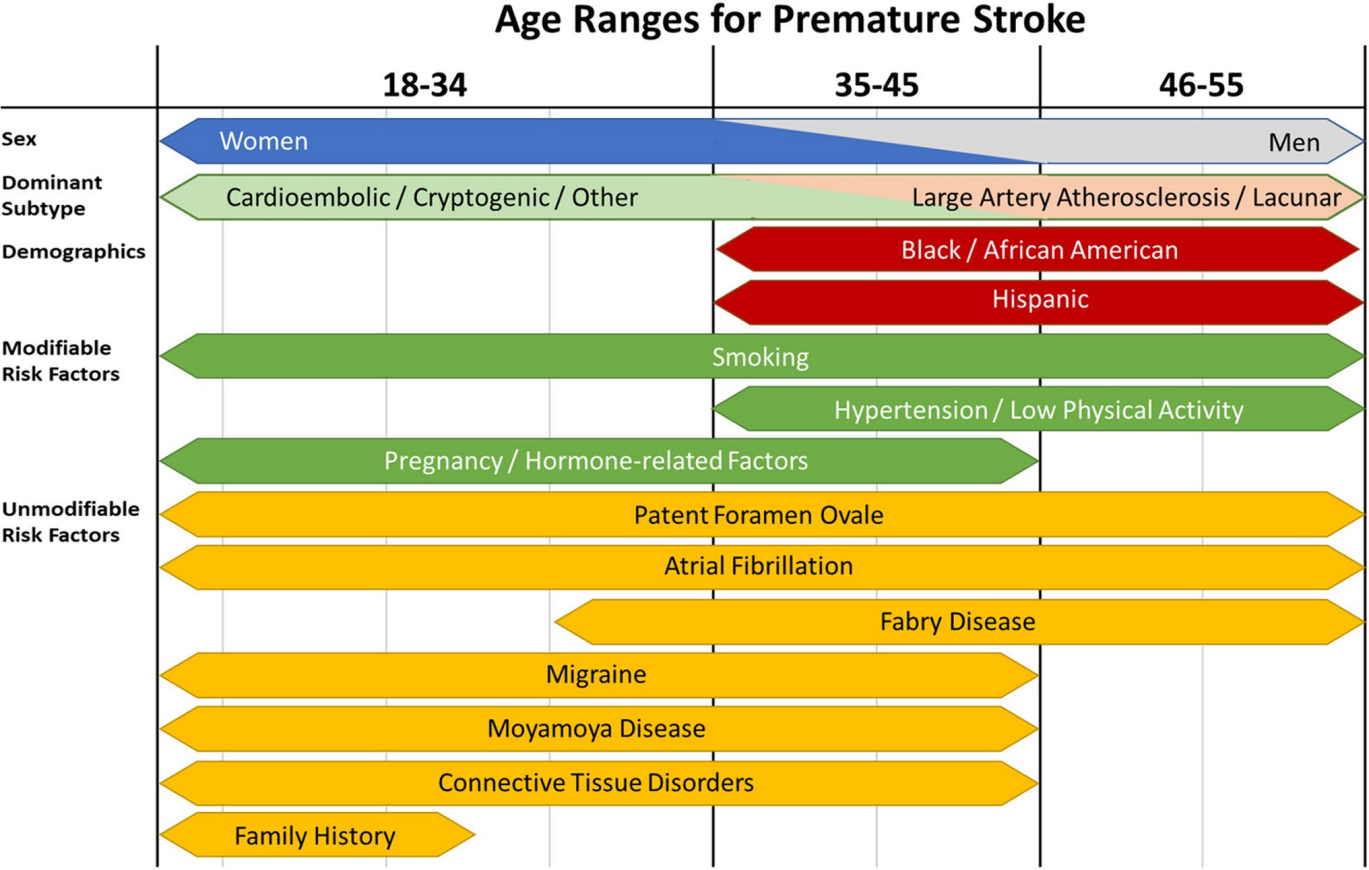
Overview of major risk factors and characteristics of premature stroke across the age ranges where they are most prominent

The modifiable risk factors associated with premature IS are similar to those among older populations. Primary risks across the young age range include hypertension, smoking, low physical activity, and hyperlipidemia [44]. Trends additionally demonstrate increased stroke incidence associated with growing cumulative comorbidity burden, even at this younger age. An overview of risk factors and premature stroke characteristics is provided in Fig. 1.

### Modifiable Risk Factors

While the overall distribution of young IS etiologies may differ from typical age groups, the vascular risk factors that accumulate over time are more consistent with older cohorts. The first and perhaps most singular vascular risk for young patients is smoking. Current smoking among the premature cohorts is highly common, appearing in up to 44% of young IS patients, while it only appears to be linked with ~ 24% of older strokes [45, 46]. This relationship is dose-dependent—an increase in smoking behaviors yields corresponding increases in the development of atherosclerotic and cardioembolic strokes [46, 47]. Further, the risk posed by smoking is not self-contained and noticeably interacts with other risks, including oral contraception and migraine, to compound total risk of IS [48, 49].

Much like smoking, hypertension and low physical activity present a considerable risk for increased IS risk [50]. These risks appear to be greater in men and increased in the 35–44 and 45 + age ranges, aligning with the shift in stroke risk from women to men [12, 14, 17•, 44]. Although it may be less common in young patients, the risk incurred by hypertension is noticeably higher than in older patients [51], possibly owing to reduced recognition and treatment among the young [52•]. Investigations into obesity have yielded contrasting results. Some recent evidence suggests that obesity portends greater risks of IS within the young adult population with increasing body mass index yielding incremental increases in IS hazard [53–55]. However, other studies have found no significant association between obesity and stroke risk after controlling for other pertinent variables [55]. Dyslipidemia represents another significant vascular risk factor commonly tied to premature IS, with higher incidence in men [12, 56] and even among children [57]. Despite this common association, the overall mechanism linking lipid profiles to stroke remains somewhat uncertain [58].

This apparent cluster of vascular risk factors is highly interconnected, with smoking contributing to hypertension and hyperlipidemia and a marked association between hypertension and hyperlipidemia [59, 60]. Notably, however, each of these factors has shown an increased prevalence among males and generally provides the greatest impact in the 35–55 age cohorts, where stroke is more common among men [12, 56]. Young women may be exposed to a separate set of risks. Pregnancy stands out as a clear gender-specific risk within younger age groups; however, current evidence links it to < 5% of strokes among young women [13, 15]. Oral contraception and hormone replacement therapy also increase stroke risk via thromboembolic mechanisms [11, 13, 61]. Recent meta-analysis has suggested a dose and time-dependent relationship between oral contraception and stroke, with every 10-µg dose increase and each additional 5 years of usage increasing IS risk by 20% [62]. These factors provide notable, age-dependent risks for women that contribute to the increased female stroke prevalence within the youngest age groups.

### Genetic and Unmodifiable Risk Factors

From the current discussion, we can see that modifiable risk factors associated with young IS broadly group into hypertension-related and pregnancy/hormone-related groups. While these stand as independent risks, they may augment the risk associated with genetic factors and chronic diseases. Since a considerable number of IS in the young are attributed to cryptogenic or other etiologies, there may be several underlying conditions contributing to IS risk. Particular conditions of interest are PFO, migraine with aura, atrial fibrillation (Afib), Fabry disease, Moyamoya, and connective tissue disorders.

Patent Foramen Ovale is relatively common. Most estimations suggest that it may appear in about 27% of the general population with variably reported sex differences [15, 63, 64]. Incidence in patients with cryptogenic stroke is much higher, and it has been found in up to 62.6% of young strokes of unknown etiology [35, 39]. Furthermore, PFO also interacts with pregnancy and other thrombotic risks [65]. Given the relatively high prevalence of PFO in the general population, its true causality with IS among the young is a matter of current debate [66]. Nevertheless, observational data report PFOs to be related to stroke in patients without other risk factors [63], and surgical closure may reduce recurrent stroke risk among young patients. While both PFO closure and anticoagulation appear to reduce IS, the use of long-term anticoagulation at a young age may increase risk of bleeding disorders [67, 68].

Similar to PFO, Afib presents an increased risk for embolism through the disruption of regular blood flow, likely compounded by other larger systemic abnormalities [69]. There is evidence that PFO contributes to atrial vulnerability, increasing the odds of arrhythmias [70]. Within the context of young stroke, Afib rates are much lower; however, it still presents a noteworthy risk for IS [71]. Similar to older populations, diagnostic challenges of Afib, primarily due to paroxysmal episodes, may result in its underreporting [72]. An association has also been identified between Afib and migraine with aura, compared to migraine without aura [73]. Migraine, in itself, also presents an increased risk for cardioembolic stroke, with vascular dysfunction identified in migraneurs [48]. The overall prevalence of migraine with aura is higher in young women, and the present IS risk is compounded by other factors such as smoking and the use of oral contraception [49, 74].

Fabry disease is a lysosomal storage disorder that leads to thickening within larger vessels [75]. While rare (1 per 100,000) in the general population, 24–48% of patients with Fabry disease experience an incident stroke, particularly at a younger age (28–54 years) [76, 77]. Whereas Fabry disease is a hereditary trait, the cause of Moyamoya disease is uncertain. Moyamoya, which causes progressive narrowing of cranial arteries, appears primarily in people younger than 50 [4••] and is more frequent in low income and urban populations. Risks also appear increased among women, people aged 18–44, and Asian/Pacific Islanders, with high prevalence specifically noted in Japanese cohorts. [78, 79]. While Moyamoya is more strongly associated with risks of IS, it also presents an increased risk of hemorrhage [80].

Just as PFO is linked to cryptogenic stroke, and Fabry Disease and Moyamoya are associated with atherosclerosis, connective tissue disorders may contribute to cervicospinal dissection. A number of conditions may be assessed here, including Ehlers-Danlos syndrome, fibromuscular dysplasia, and Marfan syndrome [35]. These represent genetic disorders that increase the fragility of blood vessels and subsequent stroke risks [35, 81]. This increases the risks associated with trauma and may lead to cervical artery dissection. While options are limited to improve these risks, recent opinion highlights a need to assess possible traumatic triggers in young patients with potential stroke [82].

It should be noted that the risk of premature stroke carries a heritable component even in the absence of recognized genetic conditions. Results from the Framingham Heart Study indicate that the risk of IS was more than doubled among children whose parents had history of premature stroke (< 65 years) [83], whereas genome-based variability only explains around 38% of IS risk [84]. While collective evidence directly assessing the contribution of family history to stroke among the young is limited, results do suggest that premature IS patients are more likely to have a positive family history than older patients [85], and family history carries the strongest association within the youngest age group (15–24 year) compared to 25–34 or 35–49 year subgroups [86]. This heritability also appears constant across IS subtypes [85].

## Social Determinants of Health

Coinciding with the geographic and racial disparities, socioeconomic factors and social determinants contribute significantly to both the incidence and outcomes of IS [87]. An important note should be made regarding potential disparities between urban and rural populations among the young. Extending from what we know about older populations and disparities within risk factors [88], it seems likely that young IS rates would be similarly elevated. Considering that social isolation and influence of social determinants increase IS incidence among older populations [89, 90], it may be expected that such social factors would carry similar impact in young cohorts. Direct studies are limited; however, current reports have found that young patients who achieved a favorable post-IS outcome (modified Rankin Scale [mRS] score of 0–1) were more likely to be college educated and practice sports [91]. Studies of premature heart disease have shown elevated burdens of risk factors, including those with important links to premature IS, in patients with low socioeconomic status. Furthermore, patients with higher sociodemographic risks are less likely to be aware of stroke symptoms [52•]. These factors may be further compounded by access and quality of care disparities, as higher socioeconomic status patients are more likely to receive high quality in-hospital and rehabilitative care [87]. This remains a topic with limited evidence and a high potential for future research.

## Diagnosis and Treatment

In part due to their rarity, premature strokes carry an increased chance of misdiagnosis, particularly among patients under 35 and those with posterior circulation strokes [92]. Studies report headache and peripheral vertigo as the most common symptoms resulting in misdiagnoses of posterior circulation strokes. However, such misdiagnoses may also be attributable to emergency medical staff as opposed to neurologist-based assessments [92, 93]. Up to 50% of young adults with apparent stroke-like symptoms may not have a stroke mimic [94]. Thus, diagnostic accuracy is exceedingly critical among young patients with stroke symptoms, and expanded utilization of magnetic resonance imaging (MRI) to confirm suspected stroke should be considered [95].

Similar to stroke among older adult populations, management of premature strokes is driven by specific stroke types and etiologies [96]. In the acute IS phase, thrombolysis and reperfusion strategies remain the most effective options [94]. Given the varied etiology, a more thorough diagnostic workup for young IS patients may be warranted. Further management is directed by objectives of avoiding complications and promoting recovery. Secondary stroke prevention strategies would be overlapping with stroke in older adults but are largely driven by specific stroke etiology. For example, following dissection, antithrombotic (antiplatelet or anticoagulant) modalities require consideration [97]. Use of statins after IS is also associated with lower rates of mortality and decreased stroke recurrence [98]. Despite established treatment and secondary prevention strategies remaining effective for younger populations, young patients are more likely to experience delays in contacting emergency services, seeking appropriate care, and receiving accurate diagnosis, which hinders treatment and jeopardizes recovery [92, 94, 99]. The interplay of socioeconomic factors with delay or suboptimal care also requires special consideration. For example, disadvantaged patients tend to have longer reperfusion times [100]. Perhaps the most effective avenue to reduce the burden of premature strokes is through development of targeted primary prevention strategies, implemented across a robust public health infrastructure. These may include cessation of smoking, awareness and appropriate treatment of hypertension and dyslipidemia, and improved diet and lifestyle choices.

## Outcomes

Premature strokes are associated with lower mortality as compared to older stroke patients. However, in-hospital death remains a significant risk, occurring in ~ 5–8% of premature ischemic and 12–34% of premature hemorrhage strokes [42, 101–103]. Mortality rates for IS are estimated at 10% within 5 years, increasing to 27% within 20 years [104].

In general, younger patients exhibit better functional recovery and outcomes than older groups [105], with higher rates of younger patients achieving mRS scores of 0–1 or 0–2 at follow-up [11, 106]. This may partly be due to an increase likelihood of receiving rehabilitative care [105]. Additionally, despite early benefits, a prospective study demonstrated that 20-year mortality rates among premature stroke patients are significantly higher than the general populace when matched for age, sex, and calendar-year [104].

Despite lower mortality, even mild impairments have been shown to result in a substantial decrease in quality of life and lost DALYs. Poor functional outcome, defined as mRS scores 3–6, were recorded in 6–20% of young stroke patients [11].

An Australian study reported an average 5-year economic burden of approximately $150,000 per stroke patient, with patients experiencing a loss of 3.05 Quality Adjusted Life years (QALY) over 5 years post-stroke and 14.22 QALYs lost over 30 years [107]. Based on data from 18–64-year-old cohorts, the cost of stroke in terms of lost DALYs is also expected to be higher in developing countries [108]. Other studies have reported that young post-stroke patients also show persistent disabilities such as significantly slower walking speeds, which is strongly associated with the ability to return to work [109]. This, paired with long-term persistent cognitive impairments experienced by up to 50% of young stroke patients [110, 111] and a number of psychosocial dysfunctions (anxiety, depression, etc.), markedly diminishes quality of life [112]. Collectively, these consequences introduce prohibitive costs for young stroke patients.

## Gaps in Knowledge and Further Research

Large gaps remain in our understanding of premature stroke. Dyslipidemia is a recognized and significant risk among young adults; however, its causal mechanism is not completely understood. Knowledge regarding the apparent heritability of premature strokes is also lacking. Atrial fibrillation and PFO, while both recognized as risk factors, have open questions regarding their true prevalence in the population.

On a population level, research effort is also needed to investigate potential socioeconomic and treatment disparities and how these may feed into the experience of stroke and recovery. This is true in both domestic and global contexts, as data are lacking for many diverse countries and populations [4••]. Concordantly, awareness of stroke risks and symptoms is lower among minorities, and disadvantaged communities and improvement of these may reduce disparity. Providers, too, may benefit from additional training or the use of MRI to improve diagnostic accuracy. Moreover, while trends and patterns have emerged among young stroke patients, little effectively explains the recent rise in premature stroke incidence.

Finally, respiratory infection has been shown to increase risks of atherothromboembolic and cardioembolic stroke [113, 114]. As we continue to investigate the consequences of the global COVID-19 pandemic, emergent information suggests that COVID-linked IS occurs most often within the presence of other comorbidities [115]. It is plausible that the increasing comorbidity burdens of young adults, paired with reduced activity due to COVID restrictions, may further increase the relative risk of premature stroke.

## Conclusion

There is now ample evidence of increasing incidence for ischemic and hemorrhagic stroke in the young. The population experiencing premature stroke is heterogeneous in terms of epidemiology, etiology, and modifiable and unmodifiable risk factors. Generally, the youngest group of patients contains a higher proportion of women and is more likely to be coagulopathic in nature. On the other hand, as age increases, premature stoke patients show a gradual buildup of traditional vascular risk factors and begin to etiologically resemble strokes encountered at older ages. Astute clinical exam and low threshold for diagnosis are necessary. However, the cornerstone of reducing young stroke burden is to be driven by primary prevention strategies implemented across a strong public health infrastructure. Such measures particularly need to address systemic sources of health inequity and disparity.
